# Racial Differences in Biopsychosocial Pathways to Tobacco and Marijuana Use Among Youth

**DOI:** 10.1007/s40615-024-02035-8

**Published:** 2024-05-28

**Authors:** Shervin Assari

**Affiliations:** 1 Department of Internal Medicine, Charles R Drew University of Medicine and Science, 1731 E. 120th St., Los Angeles, CA 90059 USA; 2 Department of Family Medicine, Charles R. Drew University of Medicine and Science, Los Angeles, CA USA; 3 Department of Urban Public Health, Charles R. Drew University of Medicine and Science, Los Angeles, CA USA; 4 Marginalization-Related Diminished Returns, Los Angeles, CA USA

**Keywords:** Socioeconomic status, Adolescents, Ethnic groups, Tobacco, Marijuana

## Abstract

**Background:**

The influence of socioeconomic disparities and multidimensional stressors on youth tobacco and marijuana use is recognized; however, the extent of these effects varies among different racial groups. Understanding the racial differences in the factors influencing substance use is crucial for developing tailored interventions aimed at reducing disparities in tobacco and marijuana use among adolescents.

**Aims:**

This study aims to explore the differential effects of socioeconomic disparities and multidimensional stressors on tobacco and marijuana use between Black and White adolescents.

**Methods:**

Utilizing longitudinal data from the Adolescent Brain Cognitive Development (ABCD) study, this research includes a cohort of pre-youth, monitored from the age of 9–10 years for a period of up to 36 months. We examined the impact of various socioeconomic status (SES) indicators and multidimensional stressors, including trauma, financial stress, racial discrimination, and family stress, alongside baseline average cortical thickness and the subsequent initiation of tobacco and marijuana use over the 36-month follow-up.

**Results:**

Overall, 10,777 participants entered our analysis. This included 8263 White and 2514 Black youth. Our findings indicate significant differences in the pathways from SES indicators through stress types to cortical thickness between Black and White youths. Notably, cortical thickness’s impact on the future initiation of tobacco and marijuana use was present in both groups.

**Conclusion:**

The study suggests that compared to White adolescents, Black adolescents’ substance use and associated cortical thickness are less influenced by stress and SES indicators. This discrepancy may be attributed to the compounded effects of racism, where psychosocial mechanisms might be more diminished for Black youth than White youth. These findings support the theory of Minorities’ Diminished Returns rather than the cumulative disadvantage or double jeopardy hypothesis, highlighting the need for interventions that address the unique challenges faced by Black adolescents.

## Introduction

Youth substance use is not distributed randomly in society and is more common in low socioeconomic status (SES) sections. As such, social epidemiologists have tried to understand this distribution of youth substance use [1]. However, SES does not directly influence substance use, and this effect may be carried through a number of processes and factors. To better understand a more holistic picture of substance use inequalities in society [2], researchers have introduced and tested several models that include an array of biopsychosocial factors [3–5]. These biopsychosocial models [6] are comprehensive frameworks that propose a sequence of chains and cascades that carry the effects of social environment to the brain and behavior [7]. They commonly start with SES indicators that reflect social context, and then they continue with the perception of the environment which is commonly measured in terms of stressors, and neurobiological changes—such as variations in cortical thickness—to shed light on their combined influence on substance use behaviors [8]. This holistic approach reveals the intricate interplay among social, environmental, neurobiological, and behavioral factors that drive substance use [9]. It captures the biological, psychological, and social dimensions that shape the experiences of young people [10]. Exploring these interactions provides a nuanced view of the origins of social and economic disparities in substance use, especially with regard to substances like tobacco and marijuana. This insight is crucial for developing more effective prevention and intervention strategies that aim to create a more equitable society [11, 12].

A wide range of SES indicators have emerged as the foundational causes of substance use [13]. Factors such as parental education, employment status, and household and neighborhood income, in addition to race and ethnicity, correlate with youth substance use [14]. These SES indicators make the larger social context in which the youth is living [15]. These SES effects, however, are in part because youth in low SES environments are more likely to experience multidimensional stress exposure, which has implications for neurobiological function [16] and cortical structure [17]. In this view, SES indicators are the most upstream factors that impact more downstream effects such as stressors on substance use [18, 19]. SES indicators are modifiable through policy change, as fairness of society investment on tax policies, educational investments, community development, and poverty prevention can change how SES is distributed across populations [20].

Stressors function as a bridge between SES indicators and neurocognitive changes and associated substance use [21, 22]. However, stress is itself heterogenous and encompass financial strain, family stress, discrimination, and life trauma [23]. Although some are more common than others, many of these stressors impact the risk of substance use [24, 25]. Many youth may turn to tobacco or marijuana to cope with various types of stress [26, 27].

As many cortical structures such as the prefrontal cortex (PFC) and the anterior cingulate cortex (ACC) [17] are shown to be involved in substance use, some researchers have used average cortical thickness as a key neurobiological mediator in the intricate web of causation from SES to substance use. Multiple cortical areas are also affected by SES and stress exposure, which explains why the cerebral cortex would have implications for youth substance use [28, 29]. Thus, it is likely that alteration in the average cortical thickness may mirror the cumulative impact of low SES and associated stressors; this may provide an efficient (despite not exclusive or complete) biological market to study biopsychosocial mechanisms that social environment gets under the skin and influences youth substance use [28, 29].

However, a growing literature on minorities’ diminished returns (MDRs) also called marginalization-related diminished returns (MDRs) suggests that the effects of SES on stress [30–33], effects of SES on brain measures such as the cerebral cortex [34–40], and effects of SES on substance use may differ by race. In MDRs framework, instead of being a biological marker, race is a proxy of racism and what race does to the individual susceptibility and vulnerability. That is, because of social stratification, segregation, and historical injustice [41–45], SES effects have weaker effects of racialized group [46–48], and Black youth who face stress on a daily basis may develop some tolerance and habituation as shown in the resilience and habituation literature [49–54]; thus, it is plausible to expect racial variation in the SES-stress-cortex-substance sue path, which is called here the biopsychosocial model of substance use.

### Aims

By knitting together social determinants, multidimensional stressors, and changes in brain structure within a unified biopsychosocial model, we enhance our understanding of the complex mechanisms driving substance use. This model not only emphasizes the importance of considering a broad spectrum of factors in substance use research but also highlights the necessity for comprehensive strategies that address the intertwined social, psychological, and biological dimensions of substance use disparities. With a focus on tobacco and marijuana—two prevalent substances among youth—this study delves into the pivotal stages of early adolescence.

## Methods

Our research involved a re-examination of data from the Adolescent Brain Cognitive Development (ABCD) study, a comprehensive longitudinal study that explores the development of preadolescent children from diverse racial and economic backgrounds as they transition into adolescence [55]. Detailed methodologies of the ABCD study have been documented extensively elsewhere [55]. The ABCD dataset is distinguished by its longitudinal scope, national reach, and the racial, socioeconomic, and geographical diversity of its sample. Schools served as the primary recruitment venues for the study participants [56]. Our analysis focused on a demographic comprising 6003 non-Latino White and 1562 non-Latino African American preadolescents. Ethical clearance for the ABCD project was obtained from the Institutional Review Board (IRB) at the University of California, San Diego (UCSD), with assent from all adolescent participants and informed consent from their parents [57].

The variables for this study encompassed race, demographic characteristics, socioeconomic factors, adversities, and substance use.

### Predictor Variables

#### Socioeconomic Status Indicators

- *Household composition*: Reported by parents, the presence of parents in the household was categorized into either single (0) or dual-parent (1) households.
- *Household income*: Derived from a 1 to 10 scale based on the ABCD study’s classification, where a higher number indicated greater income over the past 12 months, with intervals spanning from less than US $5000 to over US $200,000. This variable was treated as a continuous measure for analysis.
- *Parental education*: Inquiries about the highest level of education attained were measured on a scale from 0 (never attended school/kindergarten only) to 21 (doctoral degree), with higher values indicating greater educational achievement.
- *Neighborhood SES*: Median family income by zip code was collected and adjusted by dividing by 5000 to facilitate interpretation of beta coefficients.

### Mediator 1

#### Adversity and Stress

Baseline interviews captured adverse life experiences using a validated Life Events History instrument. The total impact of negative life events during adolescence was quantified on a continuous scale.

##### Economic-Related Strain

A 7-item scale assessed family financial stress over the past year, with questions ranging from food insecurity to inability to afford medical care. Scores were averaged to create a continuous measure of financial stress.

##### Racial Stress (Discrimination)

Experiences of discrimination were measured annually using a 7-item scale, with higher scores indicating more frequent perceived discrimination.

##### Family-Related Stress

The Family Environment Scale assessed family conflict through nine items reflecting negative familial interactions, producing a continuous score where higher values indicated greater conflict [58, 59].

### Mediator 2

- *Mean cortical thickness*: Using structural MRI, cortical thickness was borrowed from ABCD structural measure file at baseline.
- *Moderator*
- *Race:* Parent-reported race was classified into African American White, the latter serving as the reference group.

### Outcome Variable

#### Substance Use

The assessment of tobacco and marijuana initiation was conducted every 6 months. In our study, substance use referred to experimental use and initiation rather than regular use. We calculated separate variables for the new onset of marijuana and tobacco use. These variables were then combined to create a latent factor that captured their common variance. Tobacco and marijuana initiation were identified if a full cigarette (more than just a puff) was used during the follow-up period, which lasted up to 36 months post-baseline.

### Statistical Analysis

We conducted our data analysis using Stata 18.0. The first step involved providing a descriptive overview of the study variables, segmented by racial categories. Following this, we examined the correlation matrix for all variables, again separated by race. Our next step involved constructing a multigroup structural equation model (SEM) [60–62], which delineated groups based on racial identity. This SEM was designed to test our biopsychosocial framework, hypothesizing that socioeconomic status (SES) indicators could predict a latent factor comprising tobacco and marijuana consumption. This prediction was made through the mediation of various stress dimensions (mediator 1) and average cortical thickness (mediator 2). Our SEM, incorporating serial mediations, aimed to trace pathways from SES, as exogenous variables, to stressors, viewed as endogenous variables. Paths were also established from stressors to cortical thickness and directly to substance use as well. The fit of our model was evaluated using standard benchmarks, including the root mean square error of approximation (RMSEA) and the comparative fit index (CFI) [63, 64]. Given our study’s extensive sample size, we opted not to use chi-square significance as an indicator of poor model fit. This approach is commonly accepted in SEM analyses involving large samples, which can result in significant chi-square tests despite an overall good fit. Our detailed multigroup SEM [65] enabled us to identify pathways that were significant for one racial group but not for another, offering nuanced insights into the varied impacts of SES on stressors, cortical thickness, and substance use across different racial groups.

## Results

Overall, 10,777 participants entered our analysis. This included 8263 White and 2514 Black youth. Table 1 provides their descriptive data. On average, mean cortical thickness was 2.73 mm (*SD* = 0.09). Parent education was 16.5 (*SD* = 2.77) years on average. Mean age at baseline was 9.48 years (*SD* = 0.51).

**Table 1:** Descriptive data of study variables

	White *n* = 8263		Black *n* = 2514	
	Mean	Std. deviation	Mean	Std. deviation
Age	9.480	0.505	9.471	0.513
Parent education*	17.143	2.469	15.401	2.582
Income*	5.128	2.770	4.257	2.378
Neighborhood income/50,000*	0.810	0.392	0.418	0.493
Financial difficulty*	0.309	0.895	0.988	1.490
Trauma*	0.484	1.087	0.696	1.085
Discrimination*	1.152	0.366	1.359	0.600
Family conflict*	0.690	0.983	0.901	1.121
Average cortical thickness (mm)*	2.737	0.083	2.703	0.085
	*n*	%	*n*	%
Male				
No	3895	47.1	1248	49.6
Yes	4368	52.9	1266	50.4
Married family*				
No	1779	21.5	1672	66.5
Yes	6484	78.5	842	33.5
Marijuana use				
No	8007	96.9	2395	95.3
Yes	144	1.7	60	2.4
Missing	112	1.4	59	2.3
Tobacco use				
No	7914	95.8	2390	95.1
Yes	328	4.0	110	4.4
Missing	21	.3	14	.6

^*^p < 0.05

As Table 2 shows, SES indicators, stressors, average cortical thickness, and tobacco and marijuana use showed more correlations in White than Black youth. For White and Black youth, marijuana and tobacco use showed positive correlation with each other. However, tobacco and marijuana showed more correlation with SES indicators and stressors in White than Black youth.

**Table 2:** Bivariate correlations between study variables

	1	2	3	4	5	6	7	8	9	10	11	12	13
White (*n* = 8263)													
1. Age (years)	1	.015	.012	.008	.022	.029**	− .019	− .005	− .032**	.007	− .038**	.045**	.068**
2. Sex (male)		1	.000	− .012	− .004	.011	.017	− .008	.067**	.049**	− .071**	.022	.003
3. Married household			1	0.260**	.013	0.152**	− 0.205**	− 0.118**	− .078**	− .057**	.057**	− .053**	− .096**
4. Parent education (years)				1	0.371**	0.293**	− 0.259**	− .062**	− 0.147**	− .023*	.071**	− .043**	− .082**
5. Household income					1	0.135**	− 0.174**	− .058**	− .075**	− .061**	− 0.007	− .028*	− .033**
6. Neighborhood income/50,000						1	− 0.155**	− .050**	− .088**	− .015	.029**	− .036**	− .030**
7. Financial stress							1	0.148**	0.137**	0.132**	− .032**	.052**	.078**
8. Trauma								1	.056**	.083**	− .023*	.042**	.068**
9. Discrimination									1	.044**	− .018	.042**	.073**
10. Family conflict										1	.001	.032**	.037**
11. Average cortical thickness											1	− .028*	− .015
12. Marijuana use												1	0.252**
13. Tobacco use													1
Black (*n* = 2514)													
1. Age (years)	1	.031	.048*	.054**		.058**	− .002	.000	− .043*	− .034	− .072**	.054**	.038
2. Sex (male)		1	.007	.006	.011	− .017	.032	.023	0.117**	0.45*	− .070**	.029	.056**
3 Married household			1	0.320**	0.251**	0.233**	− 0.158**	− .064**	− .017	− .093**	.030	− .040*	− .016
4. Parent education (years)				1	0.565**	0.331**	− 0.157**	.006	− .056**	− 0.119**	.034	− .046*	− .006
5. Household income					1	0.302**	− 0.221**	− .028	− .080**	− 0.122**	.057**	− .061**	.017
6. Neighborhood income/50,000						1	− 0.147**	− .030	− .070**	− .085**	.022	− .049*	− .012
7. Financial stress							1	0.174**	.054*	0.274**	− .035	.044*	− .007
8. Trauma								1	− .004	0.141**	− .021	.049*	.007
9. Discrimination									1	.075**	− .030	.083**	.028
10. Family conflict										1	.006	.036	.022
11. Average cortical thickness											1	− .027	− .042*
12. Marijuana use												1	0.259**
13. Tobacco use													1

^*^p < 0.05. **p < 0.001

### Multivariable Model

**Fig. 1:**
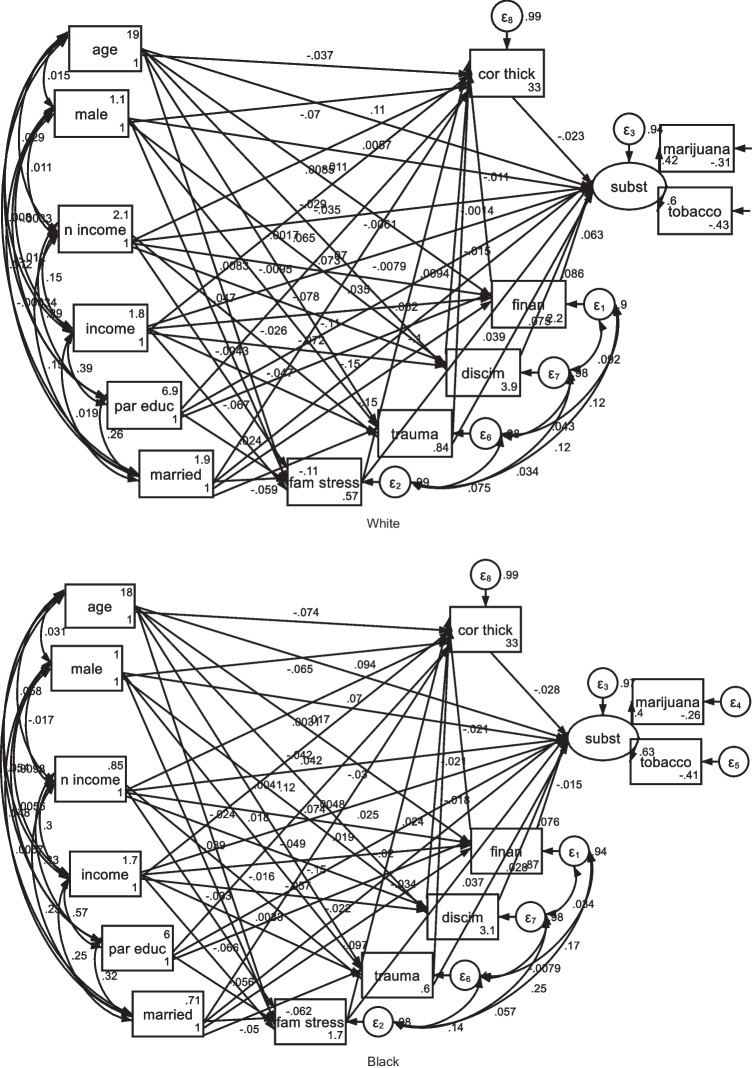
Structural equation model used for data analysis

As shown in Fig. 1 and Tables 3 and 4, our SEM analysis showed significant pathways from low SES to elevated stress across various realms, subsequently leading to less cortical thickness in White youth. Many of these associations were missing for Black youth. However, for both White and Black youth, a reduction in cortical thickness was correlated with increased youth tobacco and marijuana use. So, stress better mediated the effects of SES on substance use for White than Black youth. This model showed excellent fit: root mean squared error of approximation (RMSEA) = 0.028 (0.024–0.033), comparative fit index (CFI) = 0.959, Tucker–Lewis index (TLI) = 0.834, Akaike’s information criterion (AIC) = 210,047.129, and Bayesian information criterion (BIC) = 211,336.604.

**Table 3:** Summary of the results of the structural equation model used for data analysis (Whites)

			B	SE	95% *CI*		*P*
Structural							
Age	➔	Financial difficulty	− 0.011	0.010	− 0.032	0.009	0.279
Neighborhood income/50,000	➔	Financial difficulty	− 0.073	0.011	− 0.095	− 0.052	< 0.001
Married household	➔	Financial difficulty	− 0.149	0.011	− 0.170	− 0.127	< 0.001
Parent education	➔	Financial difficulty	− 0.152	0.012	− 0.175	− 0.128	< 0.001
Income	➔	Financial difficulty	− 0.106	0.012	− 0.130	− 0.083	< 0.001
Age	➔	Family conflict	0.008	0.011	− 0.013	0.030	0.449
Male	➔	Family conflict	0.047	0.011	0.026	0.068	< 0.001
Neighborhood income/50,000	➔	Family conflict	− 0.004	0.012	− 0.027	0.018	0.711
Married household	➔	Family conflict	− 0.059	0.011	− 0.081	− 0.037	< 0.001
Parent education	➔	Family conflict	0.024	0.013	− 0.001	0.049	0.058
Income	➔	Family conflict	− 0.067	0.012	− 0.091	− 0.043	0.000
Age			− 0.002	0.011	− 0.024	0.020	0.875
Male	➔	Trauma	− 0.009	0.011	− 0.031	0.012	0.390
Neighborhood income/50,000	➔	Trauma	− 0.026	0.011	− 0.048	− 0.004	0.023
Married household	➔	Trauma	− 0.111	0.011	− 0.133	− 0.089	< 0.001
Income	➔	Trauma	− 0.047	0.011	− 0.069	− 0.025	< 0.001
Age	➔	Discrimination	− 0.029	0.011	− 0.051	− 0.007	0.010
Male	➔	Discrimination	0.065	0.011	0.044	0.087	< 0.001
Neighborhood income/50,000	➔	Discrimination	− 0.078	0.011	− 0.100	− 0.056	< 0.001
Income	➔	Discrimination	− 0.072	0.012	− 0.095	− 0.048	< 0.001
Financial difficulty	➔	Average cortical thickness	− 0.011	0.012	− 0.034	0.013	0.374
Family conflict	➔	Average cortical thickness	0.009	0.011	− 0.012	0.031	0.396
Trauma	➔	Average cortical thickness	− 0.015	0.011	− 0.037	0.007	0.179
Discrimination	➔	Average cortical thickness	− 0.001	0.011	− 0.024	0.021	0.902
Age	➔	Average cortical thickness	− 0.037	0.011	− 0.059	− 0.016	0.001
Male	➔	Average cortical thickness	− 0.070	0.011	− 0.092	− 0.049	< 0.001
Neighborhood income/50,000	➔	Average cortical thickness	0.008	0.012	− 0.014	0.031	0.464
Married household	➔	Average cortical thickness	0.035	0.012	0.012	0.058	0.003
Parent education	➔	Average cortical thickness	0.070	0.013	0.044	0.095	< 0.001
Income	➔	Average cortical thickness	− 0.035	0.013	− 0.060	− 0.011	0.005
Financial difficulty	➔	Substance use	0.063	0.018	0.027	0.098	< 0.001
Family conflict	➔	Substance use	0.039	0.017	0.006	0.072	0.021
Trauma	➔	Substance use	0.075	0.017	0.041	0.109	< 0.001
Discrimination	➔	Substance use	0.086	0.017	0.052	0.120	< 0.001
Cortical thickness	➔	Substance use	− 0.023	0.015	− 0.054	0.007	0.128
Age	➔	Substance use	0.114	0.016	0.082	0.146	< 0.001
Male	➔	Substance use	0.006	0.017	− 0.027	0.038	0.732
Neighborhood income/50,000	➔	Substance use	− 0.006	0.018	− 0.040	0.028	0.729
Married household	➔	Substance use	− 0.105	0.018	− 0.139	− 0.070	< 0.001
Parent education	➔	Substance use	− 0.062	0.020	− 0.101	− 0.024	0.001
Income	➔	Substance use	− 0.008	0.019	− 0.045	0.029	0.674
Measurement							
Use marijuana			0.421	0.025	0.371	0.470	< 0.001
Use tobacco			0.597	0.035	0.529	0.665	< 0.001

**Table 4:** Summary of the results of the structural equation model used for data analysis (Black)

			B	SE	95% *CI*		*P*
Structural							
Age	➔	Financial difficulty	0.017	0.020	− 0.021	0.056	0.374
Neighborhood income/50,000	➔	Financial difficulty	− 0.074	0.021	− 0.115	− 0.032	< 0.001
Married household	➔	Financial difficulty	− 0.097	0.021	− 0.138	− 0.057	< 0.001
Parent education	➔	Financial difficulty	− 0.022	0.025	− 0.071	0.027	0.372
Income	➔	Financial difficulty	− 0.152	0.025	− 0.202	− 0.102	< 0.001
Age	➔	Family conflict	− 0.024	0.020	− 0.062	0.015	0.233
Male	➔	Family conflict	0.039	0.019	0.001	0.076	0.044
Neighborhood income/50,000	➔	Family conflict	− 0.033	0.021	− 0.075	0.008	0.117
Married household	➔	Family conflict	− 0.050	0.021	− 0.091	− 0.009	0.017
Parent education	➔	Family conflict	− 0.056	0.025	− 0.105	− 0.006	0.027
Income	➔	Family conflict	− 0.066	0.026	− 0.117	− 0.015	0.011
Age			0.004	0.020	− 0.035	0.044	0.838
Male	➔	Trauma	0.018	0.020	− 0.021	0.057	0.360
Neighborhood income/50,000	➔	Trauma	− 0.016	0.021	− 0.059	0.026	0.443
Married household	➔	Trauma	− 0.062	0.021	− 0.103	− 0.021	0.003
Income	➔	Trauma	0.003	0.022	− 0.040	0.047	0.881
Age	➔	Discrimination	− 0.042	0.021	− 0.083	0.000	0.048
Male	➔	Discrimination	0.118	0.021	0.077	0.158	< 0.001
Neighborhood income/50,000	➔	Discrimination	− 0.049	0.022	− 0.092	− 0.006	0.026
Income	➔	Discrimination	− 0.057	0.023	− 0.102	− 0.012	0.012
Financial difficulty	➔	Average cortical thickness	− 0.021	0.022	− 0.064	0.021	0.325
Family conflict	➔	Average cortical thickness	0.024	0.021	− 0.017	0.066	0.247
Trauma	➔	Average cortical thickness	− 0.018	0.021	− 0.058	0.023	0.393
Discrimination	➔	Average cortical thickness	− 0.021	0.022	− 0.064	0.021	0.326
Age	➔	Average cortical thickness	− 0.074	0.020	− 0.113	− 0.034	< 0.001
Male	➔	Average cortical thickness	− 0.065	0.020	− 0.105	− 0.026	0.001
Neighborhood income/50,000	➔	Average cortical thickness	0.003	0.022	− 0.039	0.046	0.887
Married household	➔	Average cortical thickness	0.019	0.021	− 0.023	0.061	0.382
Parent education	➔	Average cortical thickness	0.005	0.026	− 0.046	0.056	0.852
Income	➔	Average cortical thickness	0.042	0.027	− 0.010	0.095	0.110
Financial difficulty	➔	Substance use	− 0.015	0.032	− 0.077	0.048	0.649
Family conflict	➔	Substance use	0.037	0.031	− 0.024	0.097	0.233
Trauma	➔	Substance use	0.028	0.031	− 0.032	0.088	0.360
Discrimination	➔	Substance use	0.076	0.033	0.012	0.140	0.020
Cortical thickness	➔	Substance use	− 0.028	0.019	− 0.066	0.010	0.147
Age	➔	Substance use	0.094	0.026	0.042	0.146	< 0.001
Male	➔	Substance use	0.070	0.030	0.012	0.128	0.017
Neighborhood income/50,000	➔	Substance use	− 0.030	0.032	− 0.093	0.033	0.347
Married household	➔	Substance use	− 0.034	0.032	− 0.095	0.028	0.287
Parent education	➔	Substance use	− 0.020	0.037	− 0.093	0.054	0.597
Income	➔	Substance use	0.025	0.039	− 0.051	0.102	0.515
Measurement							
Use marijuana			0.402	0.028	0.347	0.456	< 0.001
Use tobacco			0.632	0.042	0.549	0.715	< 0.001

## Discussion

Our findings, based on longitudinal follow-up data of 9–10-year-old children, showed racial variation in the effects of low SES on tobacco and marijuana use through stress and associated changes in average cortical thickness. These mechanisms, from SES to stress and from stress to lower cortical thickness, were more applicable to White youth than to Black youth. While the effect of average cortical thickness on higher substance use was observable in both White and Black youth, the effects of stress and SES were weaker for Black youth compared to White youth. Overall, our biopsychosocial model, which includes the serial mediation of SES and stress, functioned better for White youth than for Black youth.

Although stress in youth is known as a disruptor of homeostasis, and the body’s vital equilibrium [66], stress may have differential implications for Black and White youth. As the brain develops during these formative years, the neurological, cognitive, psychological, and behavioral burden of stress may also vary by race [67]. Racial groups may differ in their appraisals of stress, and their stress response may vary across groups. While low SES and high stress are shown to have psychological impact [68, 69], these effects may vary across diverse populations. This underscores the necessity of understanding tailored programs that may help support the healthy development of diverse groups of youth.

The observation and knowledge that Black youth are more likely to experience perceived discrimination [70] and financial difficulties [71], especially compared to White youth, do not mean that they are also more vulnerable to these stressors. Still, we need anti-discrimination measures [72] and policies that reduce financial stress of all populations [73]. This disparity emphasizes how systemic racism and economic inequalities are deeply intertwined, directly affecting the mental and physical well-being of these individuals [74–78]. By prioritizing efforts to combat discrimination and by implementing policies that promote economic equity—such as targeted financial support, improved access to quality education, and job opportunities—society can address the root causes of these disparities. Such initiatives not only help in alleviating immediate financial pressures but also contribute to creating a more inclusive and equitable environment that supports the healthy development and future success of Black youth. This approach underscores the importance of structural interventions in dismantling the barriers imposed by racial and economic inequalities, highlighting a pathway toward fostering resilience and promoting fairness in the opportunities available to all individuals, regardless of their racial background.

In our study, SES was more of a precursor to stress across domains for White youth compared to Black youth. This finding suggests that variation in SES is a more salient predictor of increased stress in various domains for White children than for Black children. Although Black youth often have lower SES, their SES is less directly connected to their experiences of multidimensional stress. This aligns with previous findings that the effects of SES on stress are weaker for Black than White youth and adults [54]. These racial variations, reflecting weaker protective effects of SES, are consistent with the concept of minorities’ diminished returns, also known as marginalization-related diminished returns [36, 79–84]. This supports the intersectionality of SES and race, as proposed by other scholars, and indicates that SES indicators are not comparable across racial groups. These findings contrast with the cumulative disadvantage or double jeopardy theories, which would propose stronger vulnerability to SES for Black youth compared to White youth. Black youth are exposed to stressors such as financial instability, environmental hazards, and poor educational resources across all SES levels. Profound racial injustices in US society have diminished the relevance of high SES on the developmental trajectories of Black children compared to White children, a pattern also observed in health behaviors such as tobacco and marijuana use.

We observed a more salient mediating role of stress on cortical thickness in White youth compared to Black youth. In a previous study, we found that stress has a weaker effect as a predictor of substance use for Black youth than for White youth [85]. This may be because what is frequent may be perceived as normal, making Black individuals potentially less sensitive to stress than White individuals [85]. Black youth may have developed habituation to stress in response to the common nature of such stressors in their communities. Stress may lead to greater changes in brain structure, including cortical thinning, in youth who are less equipped to cope with it. Our observation that stress differentially mediates the relationship between SES and cortical thickness for White youth compared to Black youth supports the higher vulnerability of White youth and the higher resilience of Black youth to socioeconomic and associated adversities [86, 87]. If this is accurate, our traditional measures of stress are more suitable for predicting or explaining neurobiological changes and associated substance use for White youth than for Black youth. In other words, better measures are needed to understand the underlying neurobiological mechanisms of substance use in Black youth.

The mechanism that was similarly observed in Black and White youth was the role of average cortical thickness as a significant predictor of substance use (tobacco and marijuana) in children. This finding indicates that alterations in brain structure can influence the likelihood of engaging in substance use behaviors, regardless of race. It is particularly noteworthy that these relationships were observed in a relatively young cohort, suggesting that the foundations for substance use behaviors are laid down much earlier in life than previously assumed.

Although SES is lower and stress is higher in Black than White children, they have lower rates of tobacco and marijuana use. This presents some degrees of resilience in Black communities and families [88–92]. The weaker effects of SES and stress may be due to stronger community and family bonds, religiosity, cultural norms that discourage substance use, and perhaps a heightened awareness of the legal and social consequences of substance use given the disparities in law enforcement. Additionally, resilience, fostered by collective experiences of overcoming adversity, may equip Black youth with the coping skills to resist peer pressure more effectively. This phenomenon underscores the importance of considering the protective factors that contribute to the health behaviors of minority youth, highlighting the need for nuanced approaches in public health strategies that go beyond the conventional understanding of risk factors [87, 93–95].

Racial variation in the linkage from SES to stress and from stress to cortical thickness underscores the importance of race-based tailored programs and interventions. Policies aimed at increasing SES and reducing stressors could play a more salient role in preventing the neurological changes that predispose White children more than Black children to substance use. Furthermore, this study highlights the need for interventions that specifically address stress reduction and coping mechanisms in children from lower SES backgrounds.

This study opens several avenues for future research. Longitudinal studies that track sources of racial variation in developmental trajectories of substance use from youth into adulthood can provide further insights into the long-term effects of early low SES, high stress, and associated cortical changes. Additionally, research exploring the potential reversibility of stress-induced cortical thinning through interventions could offer hopeful prospects for mitigating the risk of substance use. Finally, examining the role of protective factors, such as supportive familial and community environments, could inform the development of comprehensive prevention strategies.

## Conclusion

In conclusion, our findings enhance the understanding of racial variation in the multifactorial biopsychosocial pathways that may contribute to substance use among Black and White children. By demonstrating the sequential relationship between SES, stress, cortical thickness, and substance use in White but not Black youth, this study underscores the critical need for tailored interventions that address the root causes of substance use across racialized populations. Such efforts are essential for preventing the neurobiological and behavioral consequences of factors that collectively contribute to inequalities in tobacco and marijuana use across diverse groups. While low SES and high stress play a significant role for White populations, a broader range of factors beyond low SES and high stress may contribute to substance use in Black youth.

## Data Availability

The ABCD data are available at the NIH NDA website: https://nda.nih.gov/abcd/.

## Code Availability

Codes are available in the appendix.
